# Single-Item Screening for Agoraphobic Symptoms: Validation of a Web-Based Audiovisual Screening Instrument

**DOI:** 10.1371/journal.pone.0038480

**Published:** 2012-07-23

**Authors:** Wouter van Ballegooijen, Heleen Riper, Tara Donker, Katherina Martin Abello, Isaac Marks, Pim Cuijpers

**Affiliations:** 1 Department of Clinical Psychology and EMGO Institute, VU University, Amsterdam, The Netherlands; 2 Innovation Centre of Mental Health and Technology (I.COM), Trimbos Institute, Utrecht, The Netherlands; 3 Department of Prevention (Prezens), Community Mental Health Centre GGZ, inGeest, Amsterdam, The Netherlands; 4 Department of Research, Community Mental Health Centre GGZ inGeest, Amsterdam, The Netherlands; 5 Leuphana University, Lüneburg, Germany; 6 Institute of Psychiatry, King’s College London, United Kingdom; Federal University of Rio de Janeiro, Brazil

## Abstract

The advent of web-based treatments for anxiety disorders creates a need for quick and valid online screening instruments, suitable for a range of social groups. This study validates a single-item multimedia screening instrument for agoraphobia, part of the Visual Screener for Common Mental Disorders (VS-CMD), and compares it with the text-based agoraphobia items of the PDSS-SR. The study concerned 85 subjects in an RCT of the effects of web-based therapy for panic symptoms. The VS-CMD item and items 4 and 5 of the PDSS-SR were validated by comparing scores to the outcomes of the CIDI diagnostic interview. Screening for agoraphobia was found moderately valid for both the multimedia item (sensitivity.81, specificity.66, AUC.734) and the text-based items (AUC.607–.697). Single-item multimedia screening for anxiety disorders should be further developed and tested in the general population and in patient, illiterate and immigrant samples.

## Introduction

Anxiety disorders are the most prevalent type of mental disorders, affecting about 17% of all people at least once in their lifetime [1]. They can improve with suitable psychological and/or drug therapy [2]–[3], but only one in four people with anxiety symptoms seek help [4].

A more accessible alternative to face-to-face therapy could be Internet-based interventions. These have proven to be as effective as face-to-face help for anxiety disorders [5] and they have several advantages. First, users can receive the treatment in the comfort of their homes and do not have to travel to a therapist or mental health centre. Second, even guided web-based interventions - which have been found more effective than unguided ones [6] - can take less therapist time than face-to-face help [7]–[8]. Third, the information can be presented in an attractive manner with audio, images and animations [9]. Fourth, outcomes of screening and progress monitoring can be instantly available to a health care provider, as well as to the users themselves if the programme is interactive or the screening leads to advice or redirection.

Both in research and in clinical practice, web-based therapy requires web-based measures to direct help-seekers to appropriate information, screen them for suitability for therapy, and monitor progress. Evidence suggests that web-based measures can be valid [10] as well as time-efficient and economically efficient [11], for example for assessing symptoms related to panic disorder (PD) [11].

For depression and anxiety disorders, web-based screening questionnaires are usually digital versions of paper-pencil measures, even though psychometric properties may be different when a screener is placed on the web [12], [13], [14]. Some studies revalidate existing questionnaires for online use, e.g. for panic symptoms [15], social phobia [16] and generalised anxiety [13]. The field of Internet intervention research is innovating rapidly [17] and developing web-based psychometric measures. Examples of web-based questionnaires for common mental disorders are the Internet-Based Self-Assessment Program for Depression (ISP-D) [18], the Web-Based Depression and Anxiety Test (WB-DAT) [19] and the Web Screening Questionnaire (WSQ) [20]. These questionnaires have moderate to good screening properties (sensitivity.63 to 1.00, specificity.44 to.97).

An additional advantage of the WSQ and the ISP-D is the use of single screening items. A single short item can quickly direct a user either to more elaborate items measuring symptom severity or to psycho-education [18], [20]. Single-item screening is a quick means to screen for mood and anxiety disorders and has been proven to be valid [13], [18]–[21], while taking less time than multi-item instruments. Some studies have shown single-item instruments are just as accurate as multi-item instruments [20]–[21], but, in general, it can be assumed that when an instrument gathers more data, its validity increases.

Existing web-based instruments may differ from paper-pencil questionnaires by using answers to instantly adapt the measure or the presentation of only one item per screen [22], but they are still text-based. The use of multimedia could aid the understanding of items, particularly by users who have difficulty reading. Studies have shown that the use of multimedia for educational purposes can result in better comprehension of the material presented, especially when images or animations are combined with text or narration [23]–[24].

A questionnaire that employs visual and auditory elements could be more appealing in general and also better suit people with lower reading levels. This motivated the development of the Visual Screener for Common Mental Disorders (VS-CMD) [25]. The VS-CMD is a 12-item questionnaire that screens for symptoms of anxiety disorders, depression, alcohol abuse and suicidal ideation. Its items consist of simple text, voice narration and images or animations.

The current study validates a single item of the VS-CMD which screens for agoraphobic symptoms. The data derive from a randomised controlled trial (RCT) of web-based self-help for subclinical to mild panic disorder (PD) in adults in the general population [26]. Randomisation of participants in the RCT is stratified for agoraphobic symptoms, because that condition can complicate certain elements of the self-help course, such as exposure exercises. That stratification is based on an item of the Panic Disorder Severity Scale – Self Report (PDSS-SR) [27], a validated, text-based questionnaire. Because a diagnostic interview is used, predictive validity can be established for both the PDSS-SR agoraphobia item and the VS-CMD agoraphobia item.

The aim of this study is twofold. First, we assess the validity of web-based single-item screening for agoraphobic symptoms. Second, we compare the multimedia agoraphobia item of the VS-CMD to the text-based agoraphobia items of the PDSS-SR, checking whether stratification for the RCT based on the agoraphobia item of the VS-CMD would be as valid.

**Figure 1:**
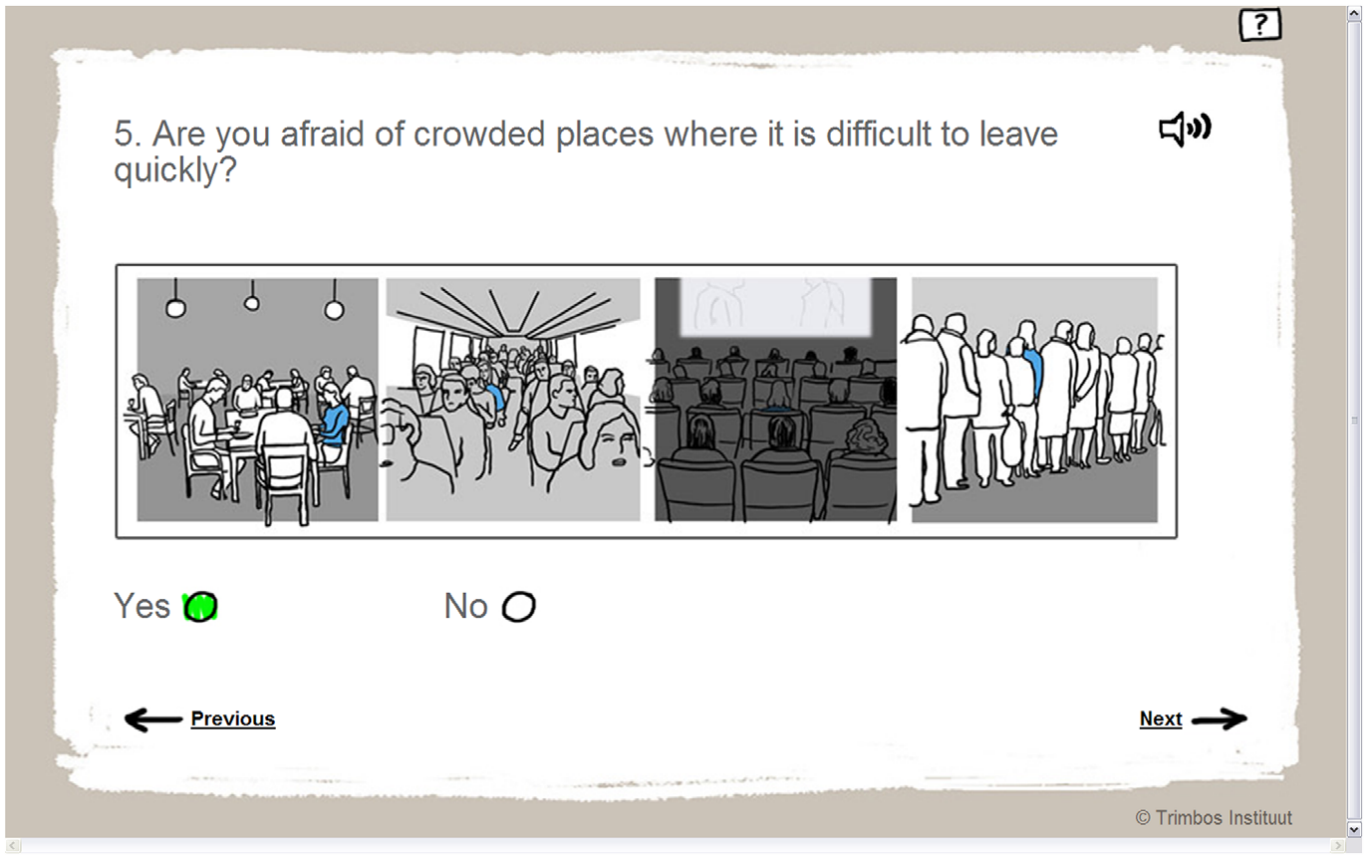
Item 5 of the VS-CMD.

## Method

### 1. Ethics Statement

The RCT protocol was approved by the Medical Ethics Review Committee of the VU University Medical Centre (METc VUmc) in Amsterdam. Written informed consent was obtained from all participants.

### 2. Participants

Participants were recruited among the general Dutch population by means of articles on a news website, a Facebook advertisement campaign, messages on panic- or anxiety-related online forums, banners on health-related websites and advertisements in newspapers. They were invited to enter a randomised controlled trial (RCT) comparing a web-based course for panic symptoms to a waiting-list control group. For more detailed information on the RCT, see the trial protocol [26]. Applicants could apply by printing and signing the informed consent form, which could then be scanned and e-mailed or returned by post. Participation required only an e-mail address and a phone number. Multiple entries by a single participant were unlikely as every participant was interviewed by phone. Data were collected from March-December 2010.

The study population consisted of adults with subclinical or mild panic disorder (PD). Inclusion criteria were: aged at least 18, Internet access, and subclinical or mild PD (PDSS-SR scores of 5–15). Individuals with too mild (PDSS-SR scores of 1–4) or too severe panic symptoms (scores of 16 or higher) were thereby excluded, as were people reporting moderate to high suicide risk. Those with severe panic or suicide risk were e-mailed advice to contact their general practitioner. The RCT was registered in the Netherlands Trial Register, part of the Dutch Cochrane Centre (NTR1639).

### 3. Instruments

#### The visual screener for common mental disorders (VS-CMD)

The VS-CMD is based on the Web Screening Questionnaire (WSQ) [20]. The WSQ is a 15-item text-based screening instrument for common mental disorders. It has proven a valid screener for social phobia, PD with agoraphobia, agoraphobia (without PD), obsessive-compulsive disorder and alcohol abuse/dependence (sensitivity .72–1.00; specificity .63–.80) [20]. Its psychometric properties were slightly more modest for major depressive disorder, generalised anxiety disorder, posttraumatic stress disorder, specific phobia and PD without agoraphobia (sensitivity: .80–.93; specificity: .44–.51). Note that these data reflect the validity of the WSQ compared to full-blown diagnoses ascertained by the CIDI by telephone [20].

The VS-CMD consists of 12 items and intends to measure clinically relevant symptoms of major depressive disorder, generalised anxiety disorder, PD, agoraphobia, specific phobia, social phobia, posttraumatic stress disorder, obsessive-compulsive disorder, alcohol abuse and suicidal ideation. Depression and suicidal ideas are each detected by two items, while other topics are detected by single items. Each item appears on a separate screen, which has been shown to be preferred by patients while being just as valid as multiple items per screen [22]. The items consist of illustrations or animations supported by a single, simply written sentence, which is also provided in spoken form. Currently, the VS-CMD is available in Dutch, English, Moroccan Arabic, Spanish and Turkish. There are male and female versions, each with corresponding images and voice-overs. The translations and voice-overs were performed by native speakers. The name “Visual Screener” has been chosen by the developers to emphasise the visual/graphic functionalities of this instrument.

The VS-CMD was compared with the WSQ among Dutch university students of ethnic Dutch and ethnic Turkish backgrounds. In the Dutch sample, the agoraphobia item did not show a significant association with its WSQ counterpart, but it did in the Turkish sample. The agoraphobia item in the Dutch VS-CMD (item 5) was therefore rephrased, without changing the graphics [25].

**Figure 2:**
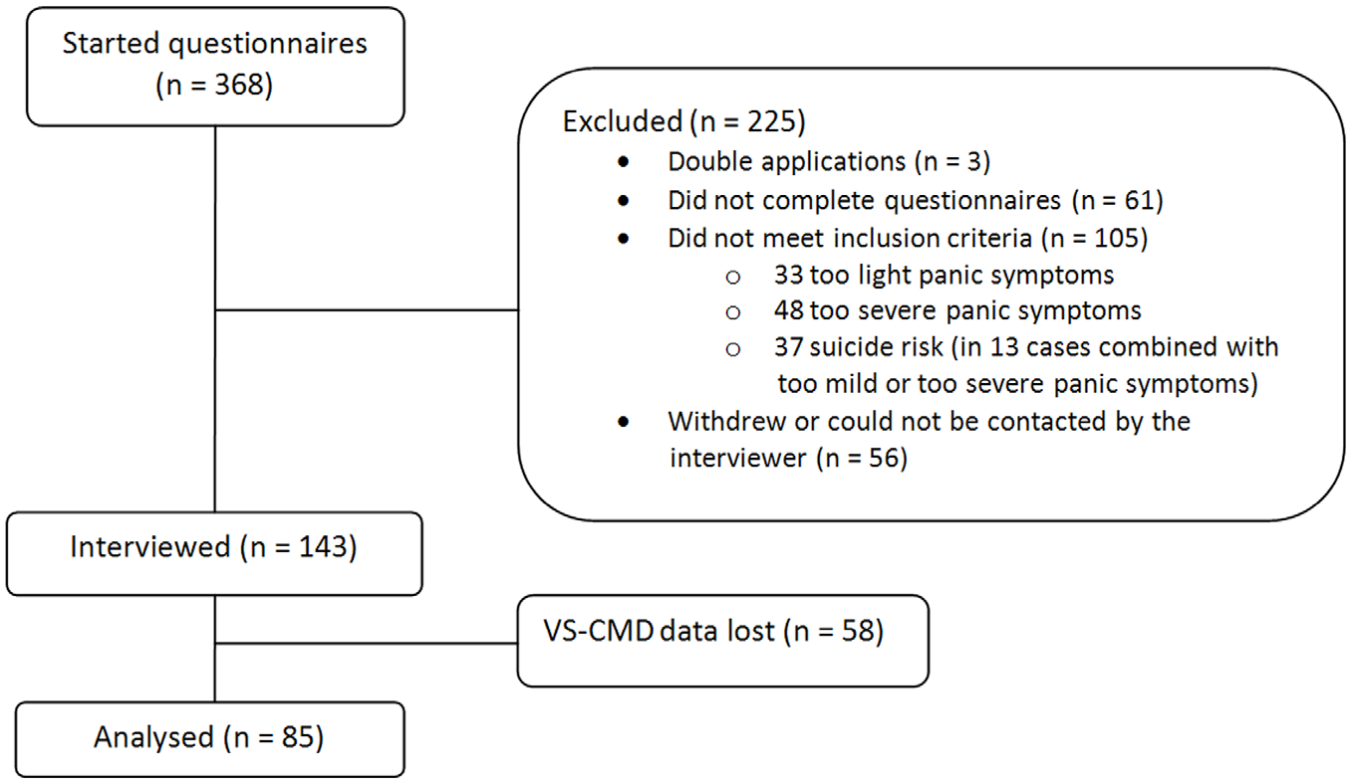
Flow chart.

**Table 1:** Demographics.

	m (SD)	*n* (%)	
Mean age	35.7 (11.1)		
Gender: female		59 (69)	
Born in the Netherlands		78 (92)	
High education (bachelors equivalent or higher)		42 (49)	
Prevalence of panic disorder (diagnosed with CIDI)		64 (75)	
Prevalance of agoraphobia (diagnosed with CIDI)		53 (62)	

*n* = 85.

Item 5 was developed to screen for clinically relevant agoraphobic symptoms, i.e. symptoms that may not meet criteria of a full-blown disorder according to the DSM-IV-TR, but are a disability for the patient and do warrant treatment. According to the DSM-IV-TR, agoraphobia means anxiety and avoidance related to places or situations from which escape might be difficult (or embarrassing) or in which help may not be available in the event of having an unexpected or situationally predisposed panic attack or panic-like symptoms [28]. The DSM-IV-TR classifies agoraphobia as subordinate to PD. It can either be diagnosed as PD with agoraphobia, or as agoraphobia without history of PD. The current paper focuses on full-blown agoraphobia that accompanies panic symptoms, first, because all of the participants suffer from subclinical to full-blown PD and second, because the gold standard (the CIDI) only makes a distinction between no diagnose and full-blown diagnose. The VS-CMD item visually depicts 4 situations that could frighten people with agoraphobic symptoms, supported by the question: ‘Are you afraid of crowded places where it is difficult to leave quickly?’ (Figure 1). The VS-CMD yields a dichotomous prediction for agoraphobic symptoms (the user answers ‘yes’ or ‘no’).

Participants in the present study completed all 12 items of the VS-CMD. A built-in clock registered how much time each participant spent on completing the instrument.

#### Panic disorder severity scale-self report (PDSS-SR)

The full PDSS-SR consists of 7 items and is a valid instrument to screen for PD with adequate psychometric properties when compared to the interview form of the Panic Disorder Severity Scale [27] [29]. Each item consists of an introduction to a specific symptom and 5 answer options scoring 0 to 4. Item 4 (PDSS-SR-4) assesses agoraphobic avoidance and item 5 (PDSS-SR-5) interoceptive avoidance, which means avoidance of situations that could induce physiological responses similar to those occurring in an anxiety crisis. PDSS-SR-5 was included in the present study, because agoraphobic behaviour associates with interoceptive avoidance. In the RCT, the stratification of the randomisation was based on the PDSS-SR-4 score alone, scores of 2 or higher denoting agoraphobic symptoms. PDSS-SR-4 correlates with measures of agoraphobic avoidance and cognitions [30]. The cut-off point of 2 is consistent with that in other studies [30], [31]. The present study used the Dutch version of the PDSS-SR [32].

**Table 2:** Agoraphobia outcomes of PDSS-SR item 4 and CIDI, cut-off  =  2. n  =  85.

	CIDI agoraphobia positive	CIDI agoraphobia negative
PDSS-SR agoraphobia positive	17	4
PDSS-SR agoraphobia negative	36	28

Sensitivity  = .32, specificity  = .88.

**Figure 3:**
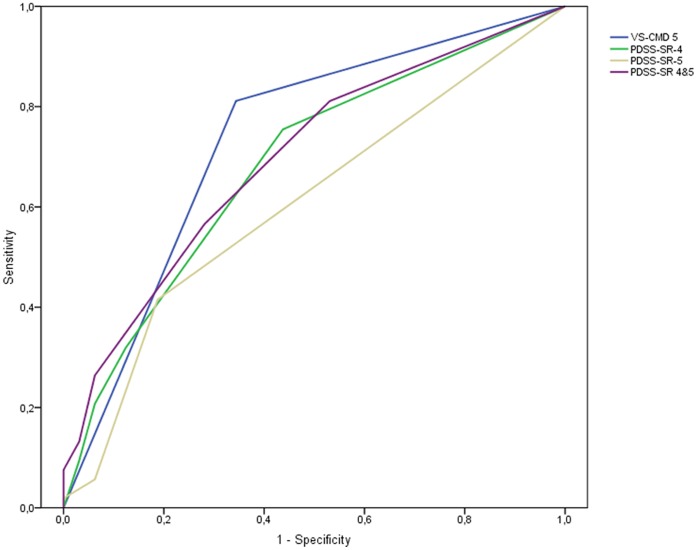
ROC Curves of VS-CMD item 5, PDSS-SR-4, PDSS-SR-5, and PDSS-SR items 4 and 5 combined. Footnote: PDSS-SR-4: AUC.684; PDSS-SR-5: AUC.607; PDSS-SR items 4 and 5 combined: AUC.697; VS-CMD item 5: AUC.734.

#### Composite international diagnostic interview (CIDI)

Diagnosis of agoraphobia was obtained using the 12-month version of the CIDI [33]–[34]. The CIDI is an extensive, fully structured and valid diagnostic interview to assess ICD-10 and DSM-IV Axis-I diagnoses [35]–[36]. The agoraphobia section of the CIDI is separate from the PD section and for the present study, only the outcome of the agoraphobia section was considered. The CIDI yields a dichotomous outcome: diagnosis or no diagnosis. The interview was administered by phone by a trained interviewer. Diagnostic interviews by telephone give highly similar results compared to face-to-face interviews [37].

#### Other variables

Other variables assessed included gender, age, nationality and education. Suicide risk was measured using 5 self-report questions derived from the Mini-International Neuropsychiatric Interview (MINI) [38]–[39].

### 4. Procedure

Candidates for RCT participation completed a battery of online questionnaires. Questions on demographic data preceded the PDSS-SR, which was followed by the questions pertaining to suicide risk. Applicants who met the criteria based on the PDSS-SR and suicide risk scores were included in the sample, and they proceeded to complete questionnaires that assessed baseline values for the RCT outcome variables. These data are not reported in this paper. Each page of the demographics and mental health questionnaires contained two or three items. The VS-CMD was filled in last, with each page displaying only one item. Subjects could go back to previous questions, but could not go forward before answering the current question. Completing all questionnaires took about 20 minutes. Data were stored digitally in a non-public database requiring a username and password to access.

Within two weeks after completing the VS-CMD and other questionnaires, each RCT participant was phoned for the CIDI and was randomly allocated immediately thereafter to the intervention group or the control group. All data used in the current paper were collected before randomisation.

**Table 3:** Agoraphobia outcomes of VS-CMD item 5 and CIDI. n  =  85.

	CIDI agoraphobia positive	CIDI agoraphobia negative
VS agoraphobia positive	43	11
VS agoraphobia negative	10	21

Sensitivity  = .81, specificity  = .66.

### 5. Analyses

First, the sensitivity and specificity of the VS-CMD agoraphobia item were calculated by comparing the participants’ scores to their CIDI diagnoses. This was also done for PDSS-SR-4. There is no consensus on which levels of sensitivity and specificity are acceptable, because those depend on the purpose, costs and benefits of the test [40]. Since the purpose of the Visual Screener is to detect clinically relevant problems, and not only full-blown disorders, sensitivity values of.70 or higher and specificity values of.40 or higher were considered satisfactory.

Second, the area under the ROC curve (AUC) was established for VS-CMD item 5, PDSS-SR-4, PDSS-SR-5 and PDSS-SR items 4 and 5 combined. The combination of PDSS-SR items 4 and 5 was tested because the combined items could have potentially yielded a larger AUC than the individual items. The AUC is a measure of validity established by plotting a measure’s sensitivity against 1 – specificity. It can range from.500 (worthless test) to 1 (perfect test). Areas under the curve of .500–.700 are said to reflect low accuracy, .700–.900 moderate accuracy and .900–1.000 high accuracy [41]. Differences between the AUCs of the different items were calculated using the formula of Hanley and McNeil [42].

Other analyses included mean scores for demographics and mean time to complete the VS-CMD. For all analyses, alpha was set at.05. Analyses were conducted with SPSS for Windows, version 17.

## Results

### 1. Sample

Of 368 applicants for the RCT, 85 (23%) were included in the present study. Of those who completed the screening questionnaires (313), 106 did not meet the RCT inclusion criteria and 64 did not complete all questionnaires or were unavailable for the telephone interview. Data of 58 participants could not be saved or were lost due to a mistake by the company hosting the VS-CMD database. See figure 2 for a flow chart. Of the 85 participants in the ultimate sample, 64% were female and the age range was 19 to 60 (M  =  35.7, SD  =  11.1). Most (92%) were born in the Netherlands and 49% had academic degrees (equivalent of bachelor’s or higher) (table 1).

### 2. Predictive Validity of PDSS-SR

In the CIDI interviews, 53 participants were diagnosed with agoraphobia. At a cut-off score of 2, PDSS-SR item 4 screened 21 participants positively for agoraphobic symptoms, corresponding to a sensitivity of.32, a specificity of.88 and an AUC of.684 (95% CI: .568–.810; see table 2 and figure 3). The optimal cut-off point for PDSS-SR-4 was 1, which has a sensitivity of.76 and a specificity of.56. Item 5 of the PDSS-SR predicted agoraphobia poorly, with an AUC of.607 (95% CI: .484–.730; figure 3); its optimal cut-off was 1, with a sensitivity of.42 and a specificity of.81. Combining item 4 and 5, predictive validity was slightly improved to an AUC of.697 (95% CI: .583–.810; figure 3).

### 3. Predictive Validity of VS-CMD

The VS-CMD agoraphobia item identified 54 participants as reflecting possible cases of agoraphobia. The sensitivity of the VS-CMD agoraphobia item was.81, the specificity.66 and the AUC.734 (95% CI: .619–.849), indicating moderate accuracy [41] (see table 3 and figure 3). The VS-CMD item predicted agoraphobia slightly better than PDSS-SR-4 (agoraphobic avoidance), although this difference (mean difference  =  0.05) was not significant (*z*  =  0.61, *p*  = .27). PDSS-SR-5 (interoceptive avoidance) yielded a lower AUC than the VS-CMD, but this difference (0.127) was short of significance (*z*  =  1.57, *p*  = .06).

### 4. Completion Time for VS-CMD

The entire VS-CMD was completed by 66% of the participants (n  =  58) in 2 to 3 minutes. The overall average was 3.6 minutes (SD = 3.7). Completion times did not differ by gender (*t* = −.267, *p* = .79), but did slightly correlate with age (*r*  = .27, *p* = .01), with a higher age associated with a longer time to complete.

## Discussion

The audiovisual agoraphobia item of the Visual Screener for Common Mental Disorders (VS-CMD) could be a valid instrument to screen for agoraphobic symptoms. In the current sample of people with panic symptoms, it predicted the outcome of a diagnostic interview with moderate sensitivity and specificity. In terms of AUC, it performed slightly better than PDSS-SR-4, though not significantly. PDSS-SR-5 performed poorly. A combination of PDSS-SR items 4 and 5 might slightly improve the ability of the PDSS-SR to screen for (full-blown) agoraphobia. The multimedia VS-CMD item screened for agoraphobia at least as well as the text-based PDSS-SR agoraphobia items combined, despite the VS-CMD item’s minimal text and dichotomous outcome. The entire 12-item VS-CMD is a brief assessment, taking about 3 minutes to complete, which is about 15 seconds per item. These results indicate the VS-CMD could be just as valid as text-based instruments, while it should be more intelligible for people with difficulty reading due to its reliance on images and audio.

In this study, the sensitivity of PDSS-SR-4 was very low at cut-off point 2. Perhaps the cut-off point could be lowered to 1 at the expense of specificity. Wuyek et al. [29] have argued that while the PDSS-SR is a valid questionnaire, it is advisable not to rely on its single items. In the RCT from which we obtained the data for the current study, PDSS-SR-4 was used to stratify the randomisation for agoraphobia. Our results imply that VS-CMD item 5 would have been an equally good or better predictor of agoraphobia diagnosis in the sample.

The psychometric properties of the multimedia VS-CMD agoraphobia item (sensitivity.81, specificity.66 and AUC.734; 95% CI: .619–.849) were found to be comparable to those of the text-based items for the various disorders on the Web Screening Questionnaire (WSQ; sensitivity.72 to 1.00, specificity.44 to.77, AUC.65 to.82 [20]). According to the Donker study, the WSQ agoraphobia item had good sensitivity (1.00), moderate specificity (.63) and a moderate AUC (.81; 95% CI .73–.90). The VS-CMD item also has comparable psychometric properties to a 5-item pen-and-paper questionnaire, the Fear Questionnaire agoraphobia subscale [43]. In a clinical sample and at an optimal cut-off point of 6, that subscale has been found to have a sensitivity of.74 and a specificity of.72, with no AUC reported [44]. A more accurate self-report instrument for agoraphobia is the Mobility Inventory [45]–[46], especially the alone subscale. This subscale consists of 28 items and predicts the diagnosis of agoraphobia with sensitivity.87 and specificity.73. Comparisons between the VS-CMD and other questionnaires [20], [44], [46] have limited meaning here, as the instruments were tested in samples with characteristics different from those of the present sample.

Our results contained 11 false positives for the VS-CMD. That is, 11 participants clicked ‘yes’ on the VS-CMD agoraphobia item but had not been diagnosed with agoraphobia in the CIDI interviews. This might suggest that the agoraphobia item addresses a common fear that is not always associated with agoraphobia, perhaps caused by the brevity of the item. During the development of the VS-CMD, it was challenging to translate a mental disorder into a single question and images or animations. The DSM-IV-TR definition of agoraphobia contains the element of fear of panic-like symptoms, which is not covered by the VS-CMD agoraphobia item. On the other hand, the false positives could also indicate that 11 participants had sub-clinical agoraphobia symptoms that do not meet CIDI criteria for a diagnosis but could still be clinically relevant. Further research into the validity of the VS-CMD should employ continuous scale measures that gauge the severity of symptoms, enabling the VS-CMD screening items to be compared to various symptom severity cut-off points.

This study has a number of limitations. First, the VS-CMD was completed after all other questionnaires (except for the diagnostic interview). This could have biased results, as participants were already aware what kinds of symptoms were being queried. Second, PDSS-SR-4 rates agoraphobic avoidance in relation to panic attacks or fear of them, whereas VS-CMD item 5 rates agoraphobic fear. Yet the comparison between these items is valid, because all participants suffered from either panic attacks or fear of panic. Third, the 12-month prevalence version of the CIDI was used, whereas the VS-CMD and the PDSS-SR assess the current state of symptoms. This implies that the sensitivity and AUCs of both the VS-CMD and the PDSS-SR items might have been higher than the outcomes suggest. Fourth, our sample was limited to people with mild to moderate panic symptoms, and the results should therefore be generalised with caution. Samples selected for other anxiety disorders or with more severe symptoms might have yielded other outcomes. Finally, the study sample consisted of Dutch people, of which half was highly educated. Considering the VS-CMD is probably most suited for people who have difficulty reading, this is an important limitation. Nevertheless, the present results show that the VS-CMD measured what it was intended to measure.

Future research should validate the other VS-CMD items, in both patient populations and the general population, in order to obtain more widely applicable results. If the VS-CMD proves a valid instrument, it could be applied on Internet portals for mental health issues in order to direct help-seekers to appropriate information or to screen applicants on eligibility criteria for online interventions. It could also be an alternative to text-based questionnaires for people with low reading levels and/or various cultural backgrounds. Very little is known about the assessment of mental health problems among low socioeconomic, illiterate and immigrant groups, perhaps because existing psychometric instruments are difficult to understand or unsuitable in other ways [47] or because the groups are underrepresented in research samples. The Center for Epidemiologic Studies Depression Scale (CES-D) [48] has been shown by a Dutch study [49] to be a valid measure of depression among elderly Turkish and Moroccan immigrants provided the items were read aloud to participants. Research on the validity and utility of audiovisual screening for psychological problems in different social groups is limited. Validity can be established only if an instrument can be compared to another measure that has already been tested in the group being studied. The lack of suitable instruments is one reason why the VS-CMD was developed.

Multimedia screening is a promising area for future research. This study shows that screening for agoraphobia may be possible using a single multimedia item. The text-based PDSS-SR-4 may also be a valid measure. Both items could be used to screen for agoraphobia, while the VS-CMD item has the advantage that it could be administered to people who have difficulty reading. Research into the validity of web-based screening instruments and single-item screening is still scarce, and more needs to be learned about the use of media other than text to screen for anxiety disorders. Further development of multimedia screening and treatment of mental disorders should be encouraged.
